# Supplementary figures and images for: Effects of synthetic and environmentally friendly fungicides on powdery mildew management and the phyllosphere microbiome of cucumber
Source: PLoS One. 2023 Mar 8;18(3):e0282809. doi: 10.1371/journal.pone.0282809 (PMC9994715; doi:10.1371/journal.pone.0282809)

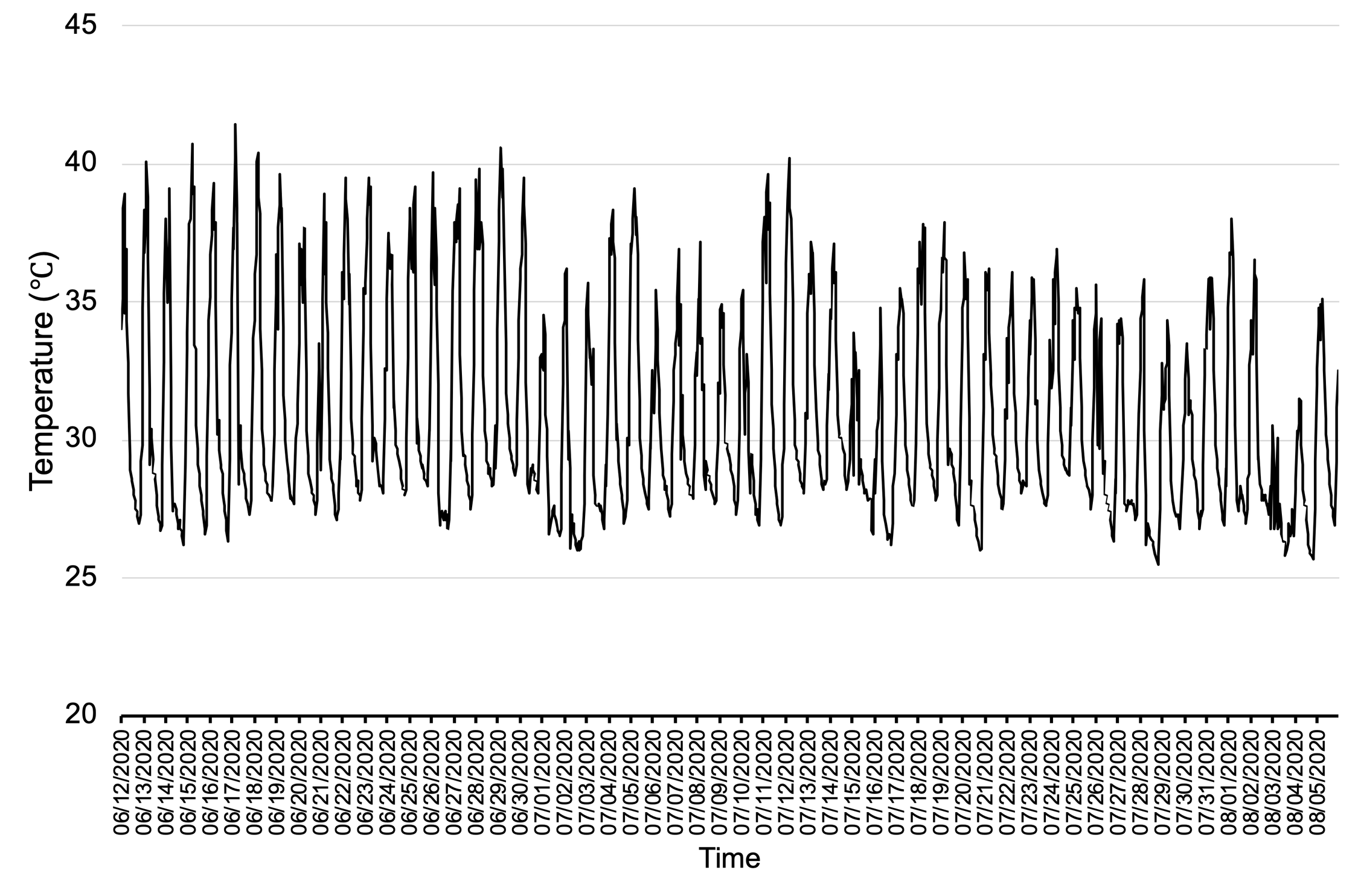

Supplement: S1 Fig — (TIF) [file pone.0282809.s001.tif]
