# Supplementary material for: Effects of synthetic and environmentally friendly fungicides on powdery mildew management and the phyllosphere microbiome of cucumber
Source: PLoS One. 2023 Mar 8;18(3):e0282809. doi: 10.1371/journal.pone.0282809 (PMC9994715; doi:10.1371/journal.pone.0282809)
Supplement: Attachment — Submitted filename: Response to Reviewer.docx [file pone.0282809.s003.docx]

**Response to Reviewer I**

Reviewer #1: The paper overlooked previous data on phosphates as inducer of resistance against powdery mildew as follows

Reuveni, M.; Agapov, V.; Reuveni, R. Suppression of cucumber powdery mildew (Sphaerotheca fuliginea) by foliar sprays of phosphate and potassium salts. Plant Pathol. 1995, 44, 31–39.

19. Reuveni, M.; Reuveni, R. Efficacy of foliar sprays of phosphates in controlling powdery mildews in field-grown nectarine, mangotrees and grapevines. Crop Prot. 1995, 14, 311–314.

20. Reuveni, M.; Agapov, V.; Reuveni, R. A foliar spray of micronutrient solutions induces local and systemic protection against powdery mildew (*Sphaerotheca fuliginia*) in cucumber plants. Eur. J. Plant Pathol. 1997, 103, 581–588

Reuveni, R.; Reuveni, M. Foliar-fertilizer therapy—A concept in integrated pest management. Crop Prot. 1998, 17, 111–118.

Response: Thank you for your suggestion. We have reviewed these articles and cited them in the manuscript. [Reference 14,15,16,18]

**Response to Reviewer II**

Reviewer #2: 1. Extracted DNA of cucumber leaves may contain the genomic sequences of both phyllosphere microbiome and endophytes (especially bacteria and fungi), then how do you distinguish the difference between phyllosphere and endophytic species through your analysis?

(In line 344 authors have mentioned about *Cladosporium* as one of the major endophytic fungi in cucumber leaves). All the genera identified in this study are phyllosphere residents or even contain endophytes? Kindly, shed some light on this.

Response: Thank you very much for the question. The method we used in this study cannot distinguish between leaf epiphyte and endophyte, so we use the term “phyllosphere microbiome” to indicate the microbiome of the whole leaves. (Bashir et al. 2022. <https://www.sciencedirect.com/science/article/pii/S0944501321001944>). Due to the restriction of the experiment method, we can only speculate whether the microorganism is endophytic or epiphytic according to previous reference. The description of *Cladosporium* is according to a previous survey of endophyte in cucumber leaves (Huang et al. 2020).

Thank you for your suggestion; we noticed that the description was too arbitrary. We have rephrased the sentence. [line 358-360]

2. If endophytes are present then what is the impact of these fungicides on the endophytic diversity present in cucumber leaves as that of phyllosphere microbiome?

**Response:** Thank you very much for the question. Follow by question 1, due to the restriction of the method, we cannot distinguish the impact of the fungicides on epiphyte and endophyte. However, since we found no difference in phyllosphere bacterial population among the three fungicides treatment, we can speculate that the fungicide did not affect epiphytic and endophytic bacteria. For the fungal population, NPA and sulfur treatment may have little effect on endophytic fungus because only a few OTUs were altered after the treatments. Tebuconazole may affect more endophytic fungus because the fungicide can be uptake by plant tissue, and some previously reported endophytic genera were found to be altered after the treatment.

3. Species level identification would enhance this study in understanding role of different bacterial species and fungal species in disease management in cucumber.

**Response:** Thank you for your suggestion. We have considered extending the analysis to the species level. However, since the primer we used only amplified the V3-V4 region for bacteria and partial ITS for fungus, the information is insufficient to classify these organisms to species level (especially for fungus). Therefore, we remained our analysis and discussion only at the genus level. In the future study, we will use 3^rd^ generation 16S and ITS full-length sequencing to improve the resolution of taxonomy classification.

4. Sulfur is best used by applying it in advance of any outbreak of powdery mildew to protect against the disease, how your study has proven it to be more effective and safe?

**Response:** Thank you for your question. In this experiment, we applied the fungicides three times after flowering [line 133]. The disease severity was low before the first spraying, so the fungicides were applied in advance of any outbreak of powdery mildew. We have investigated the disease incidence and severity of the three treatments in the manuscript to demonstrate the efficacy of different treatments (Fig 1). The result showed that both indexes of the sulfur treatment were significantly lower than the control but no statistical difference with the NPA and tebuconazole treatment. Therefore, the result can not prove that sulfur was more effective than the other two fungicides. In the manuscript, we stated that the environmentally friendly fungicides "maintained the same effect as the tebuconazole treatment".

For the safety of sulfur: sulfur is an element that exists in plant tissue and can be used in organic agriculture, so it is seen as a safety fungicide [line 86-88]. In addition, we have done the pesticide residue test [line 153-158], and no fungicide was detected in the sulfur treatment. According to these results, sulfur maintains the same effect as the synthetic fungicide but is safer, so it can replace the synthetic fungicide to control powdery mildew on cucumber.

**Additional editing:**

To further improve the clarity of the writing, the manuscript has also been reviewed by professional native English-speaking editors. The revision has been made accordingly and the certificate is provided by American Journal Experts under verification code 5DC7-46F6-5C75-8096-8FBP.
