# Supplementary material for: Effects of synthetic and environmentally friendly fungicides on powdery mildew management and the phyllosphere microbiome of cucumber
Source: PLoS One. 2023 Mar 8;18(3):e0282809. doi: 10.1371/journal.pone.0282809 (PMC9994715; doi:10.1371/journal.pone.0282809)
Supplement: Attachment — Submitted filename: Response to Reviewer_R3S.docx [file pone.0282809.s004.docx]

**Response to Reviewer III**

Reviewer #3: Wu et al. work on “Effects of synthetic and environmentally friendly fungicides on powdery mildew management and the phyllosphere microbiome of cucumber” looks quite interesting. The work and the context are appreciable, and the results deserve publication. However, there are certain points which need to be addressed before considering it for the publication.

1. In the introduction clearly state the importance of the topic and recent examples from the literature. And in the last paragraph of the Introduction, please state your motivation, goals and objectives, the novelty of this research, and potential contribution to the literature.

Response: Thank you for the suggestion. The paragraph was revised as follows:

Page 5, lines 114-129

“Motivated by the growing demand for environmentally-friendly alternatives to synthetic fungicides for sustainable plant disease management, we have investigated the effects of one synthetic fungicide (tebuconazole) and two environmentally friendly fungicides (NPA and sulfur) on the development of cucumber powdery mildew. The dynamics of bacterial and fungal communities in the cucumber phyllosphere have also been analyzed using amplicon sequencing to explore the impacts of the fungicides on the phyllosphere microbiome. This study aimed to (1) determine the relative efficacy of NPA, sulfur, and tebuconazole in controlling cucumber powdery mildew; (2) compare the phyllosphere diversity of bacteria and fungi among different treatments; and (3) identify fungal operational taxonomic units (OTUs) affected by different treatments in the cucumber phyllosphere. To the extent of our knowledge, no other studies have been conducted to evaluate the effect of foliar spray on the cucumber phyllosphere microbiome. This study may shed light on the impacts of different fungicides on the microbial composition in the leaves, providing new insights into the ecological consequences of different fungicides on the disease management of cucumber powdery mildew.”

2. Figures are poorly prepared. The quality of the figure is low and not presentable. The resolution of the figures should be improved to maintain at least a minimum quality of 600 dpi.

Response: Thank you for the suggestion. All the figures were refined to 600 dpi and reprocessed by “PACE” to meet the PLOS ONE requirements as recommended in the journal’s submission guidelines.

3. In Page 10, Table 2 displayed alpha diversity among different treatments. Representing alpha diversity of bacterial and fungal populations among different treatments as an R-plot would be far better to understand the difference.

Response: Thank you very much for the suggestion. The information on alpha diversity in Table 2 has been represented as box plots in Fig 3.

4. In Page 9, on line 264, in the description about PERMANOVA results, the authors stated that the 264 (R2 = 0.17, P = 0.942). A clear description about these values should be included. For instance, R2 value says that the fungicide treatment accounts for only 17 % variation among the samples which is statistically not significant, as evident from the p-value 0.942. Moreover, “P” should be represented as “p”.

Response: Thank you for the suggestion. The description was revised as follows:

Page 10, lines 272-278

“In addition, PERMANOVA showed that the fungicide treatment accounted for only 17 % variation of the bacterial population among the samples, although this was statistically non-significant, as evident from the p-value of 0.942 (Table 2). However, different treatments significantly affected the fungal community on cucumber leaves, as the PERMANOVA demonstrated that the treatments accounted for 57% of the variation in the fungal population with a p-value of 0.008 (Table 2).”

Lines 285-287

“The results showed that the different treatments still significantly (p = 0.023) accounted for 45% of the variation in the fungal community after excluding *Podosphaera…”*

5. Further analyses such as identification of core microbiome from the present data, correlation analysis based network models can greatly improve the understanding and add key findings to the current conclusions.

Response: Thank you for the suggestion. Because of the limited number of replicates in each treatment, Spearman’s correlation could not be properly estimated in the network analysis. Although other analyses have been considered, the results were beyond the scope of this article. To support the conclusions of this study, figures and tables that were relevant to the main findings have been included in this article and presented according to previous reviewer advice.
